# Purely laparoscopic feeding jejunostomy: a procedure which deserves more attention

**DOI:** 10.1186/s12893-021-01050-4

**Published:** 2021-01-13

**Authors:** Hsin-I. Tsai, Ta-Chun Chou, Ming-Chin Yu, Chun-Nan Yeh, Meng-Ting Peng, Chia-Hsun Hsieh, Po-Jung Su, Chiao-En Wu, Yung-Chia Kuo, Chien-Chih Chiu, Chao-Wei Lee

**Affiliations:** 1 Department of Anesthesiology, Chang Gung Memorial Hospital, Linkou Medical Center, Guishan, Taoyuan, Taiwan, Republic of China; 2 College of Medicine, Chang Gung University, Guishan, Taoyuan, Taiwan, Republic of China; 3 Graduate Institute of Clinical Medical Sciences, Chang Gung University, Guishan, Taoyuan, Taiwan, Republic of China; 4 Department of Surgery, Keelung Chang Gung Memorial Hospital, Keelung, Taiwan, Republic of China; 5 Division of General Surgery, Department of Surgery, Chang Gung Memorial Hospital, Linkou Medical Center, No.5, Fuxing St., Guishan Dist., Taoyuan, 33305 Taiwan, Republic of China; 6 Department of Surgery, New Taipei Municipal Tu-Cheng Hospital (Built and Operated By Chang Gung Medical Foundation), Tu-Cheng, New Taipei City, Taiwan, Republic of China; 7 Department of Hematology-Oncology, Chang Gung Memorial Hospital, Linkou Medical Center, Guishan, Taoyuan, Taiwan, Republic of China; 8 Division of Hematology and Oncology, Department of Internal Medicine, New Taipei Municipal Tu-Cheng Hospital (Built and Operated by Chang Gung Medical Foundation), Tu-Cheng, New Taipei City, Taiwan, Republic of China; 9 Graduate Institute of Data Science, Taipei Medical University, Taipei, Taiwan, Republic of China; 10 Department of Nursing, Chang Gung Memorial Hospital, Linkou Medical Center, Guishan, Taoyuan, Taiwan, Republic of China

**Keywords:** Purely laparoscopic, Jejunostomy, Feeding, Enteral nutrition, Minimally invasive surgery, Enterostomy

## Abstract

**Background:**

Laparoscopic procedure has inherent merits of smaller incisions, better cosmesis, less postoperative pain, and earlier recovery. In the current study, we presented our method of purely laparoscopic feeding jejunostomy and compared its results with that of conventional open approach.

**Methods:**

We retrospectively reviewed our patients from 2012 to 2019 who had received either laparoscopic jejunostomy (LJ, n = 29) or open ones (OJ, n = 94) in Chang Gung Memorial Hospital, Linkou. Peri-operative data and postoperative outcomes were analyzed.

**Results:**

In the current study, we employed 3-0 Vicryl, instead of V-loc barbed sutures, for laparoscopic jejunostomy. The mean operative duration of LJ group was about 30 min longer than the OJ group (159 ± 57.2 mins vs 128 ± 34.6 mins; P = 0.001). There were no intraoperative complications reported in both groups. The patients in the LJ group suffered significantly less postoperative pain than in the OJ group (mean NRS 2.03 ± 0.9 vs. 2.79 ± 1.2; *P* = 0.002). The majority of patients in both groups received early enteral nutrition (< 48 h) after the operation (86.2% vs. 74.5%; *P* = 0.143).

**Conclusions:**

Our study demonstrated that purely laparoscopic feeding jejunostomy is a safe and feasible procedure with less postoperative pain and excellent postoperative outcome. It also provides surgeons opportunities to enhance intracorporeal suture techniques.

## Background

Compared to the parenteral route, oral or enteral nutrition is deemed more favorable due to its lower cost, fewer complications, and preservation of gut function [1–3]. Clinicians thus extreme their efforts to apply enteral nutrition in as many patients as possible. Patients with upper gastrointestinal tract or oropharyngeal malignancy, or patients with old cerebrovascular insult complicated with recurrent aspiration pneumonia, on the other hand, are usually intolerant to per oral feeding. Feeding jejunostomy is thus commonly performed in these patients to maintain their nutrition. For obstructed upper gastrointestinal malignancies, feeding jejunostomy also serves as a route for nutritional support during neoadjuvant chemotherapy [4–6].

Owing to the advent of minimally invasive surgery, feeding jejunostomy could also be performed under laparoscopy. Laparoscopic feeding jejunostomy was first described in 1990 by O'Regan [7]. Compared to the conventional open procedure, laparoscopic approach has inherent merits of smaller incisions, better cosmesis, less postoperative pain, and earlier recovery. With advancement of surgical equipment and laparoscopic skills, several different methods of laparoscopic jejunostomy have been developed [8–19]. However, given challenging intracorporeal suture, purely laparoscopic feeding jejunostomy without the aids of fancy suturing instrument or intricate technique has rarely been described. Furthermore, jejunostomy is believed to be an insignificant procedure by many surgeons; few studies have tried to discuss the benefits of laparoscopic jejunostomy as a result. In the current study, we described our method of purely laparoscopic feeding jejunostomy and compared its outcome with that of conventional open approach.

## Methods

### Patients

Under the approval of the Institutional Review Boards of Chang Gung Memorial Hospital (CGMH), we retrospectively reviewed patients who received feeding jejunostomy in Linkou CGMH between 2012 and 2019. The patients were divided into two groups: laparoscopic jejunostomy (LJ) group and conventional open jejunostomy (OJ) group. The recruited patients’ clinical characteristics (age, gender, body mass index, surgical indications, etc.), peri-operative variables (operative time, blood loss, concomitant procedure), and postoperative outcomes (postoperative pain scale, early and late complications) were retrieved from the prospectively collected database. Patients who did not have detailed preoperative/intraoperative clinical records, or who did not have regular postoperative follow-up were excluded from our study.

The conventional open jejunostomies (OJ) were performed by experienced surgeons in the same surgical department. Laparoscopic jejunostomy (LJ), on the other hand, was conducted by a single surgeon who had a special interest in the laparoscopic procedures. The index surgeon had received a 5-year postgraduate training as a surgical resident at Linkou Chang Gung Memorial Hospital. The surgeon received, in addition to trainings in conventional open hepatobiliary and gastrointestinal surgeries, comprehensive training in both the fundamental and advanced laparoscopic procedures including laparoscopic cholecystectomy (LC), laparoscopic appendectomy (LA), laparoscopic gastrectomy (LG), laparoscopic pancreatectomy (LP), and laparoscopic hepatectomy (LH) during the past few years. Since the index surgeon had become a board-certified gastrointestinal surgeon in 2012, the LJ also started from 2012. The index surgeon, in the meanwhile, did not operate patients in the OJ group.

### Surgical procedures of laparoscopic and open jejunostomy

**Fig. 1:**
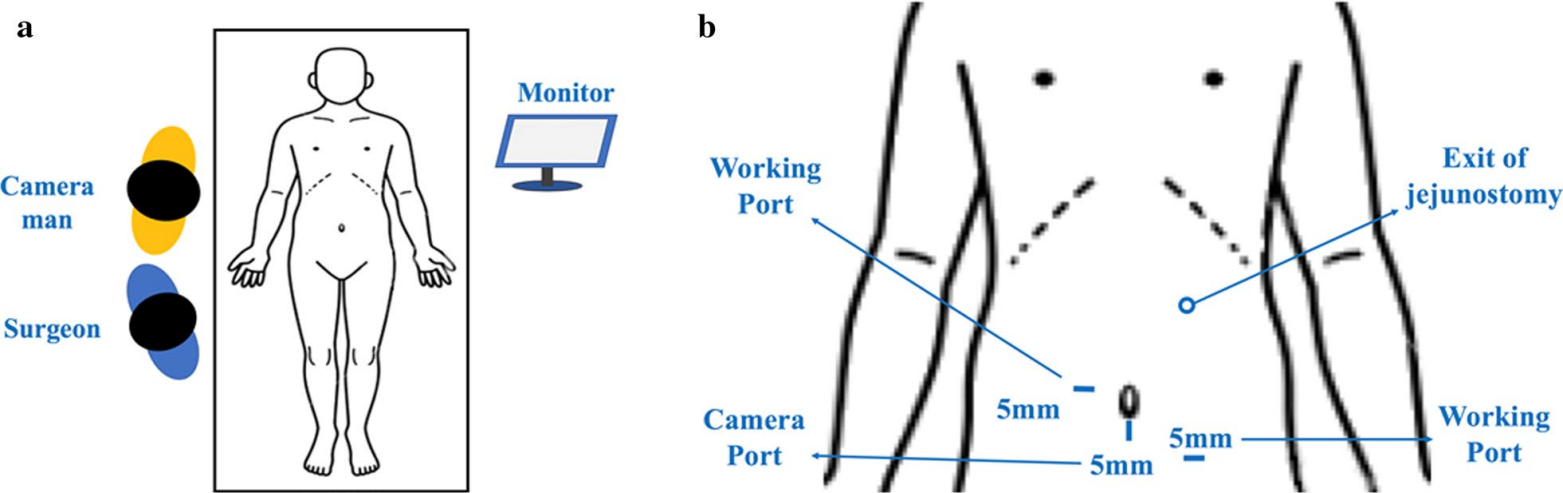
Operation room settings and the placement of trocar ports. **a** The patient was placed in supine position. The procedures were performed by two surgeons, with the index operating surgeon and camera man standing on the right side of the patient. **b** The video laparoscope was introduced via a 5 mm vertical incision at about 3 cm below the umbilicus. Another 5 mm working port was created below the umbilical level at around left mid-clavicular line. The third 5 mm working port was created slightly above the umbilical level at right paramedian area

**Fig. 2:**
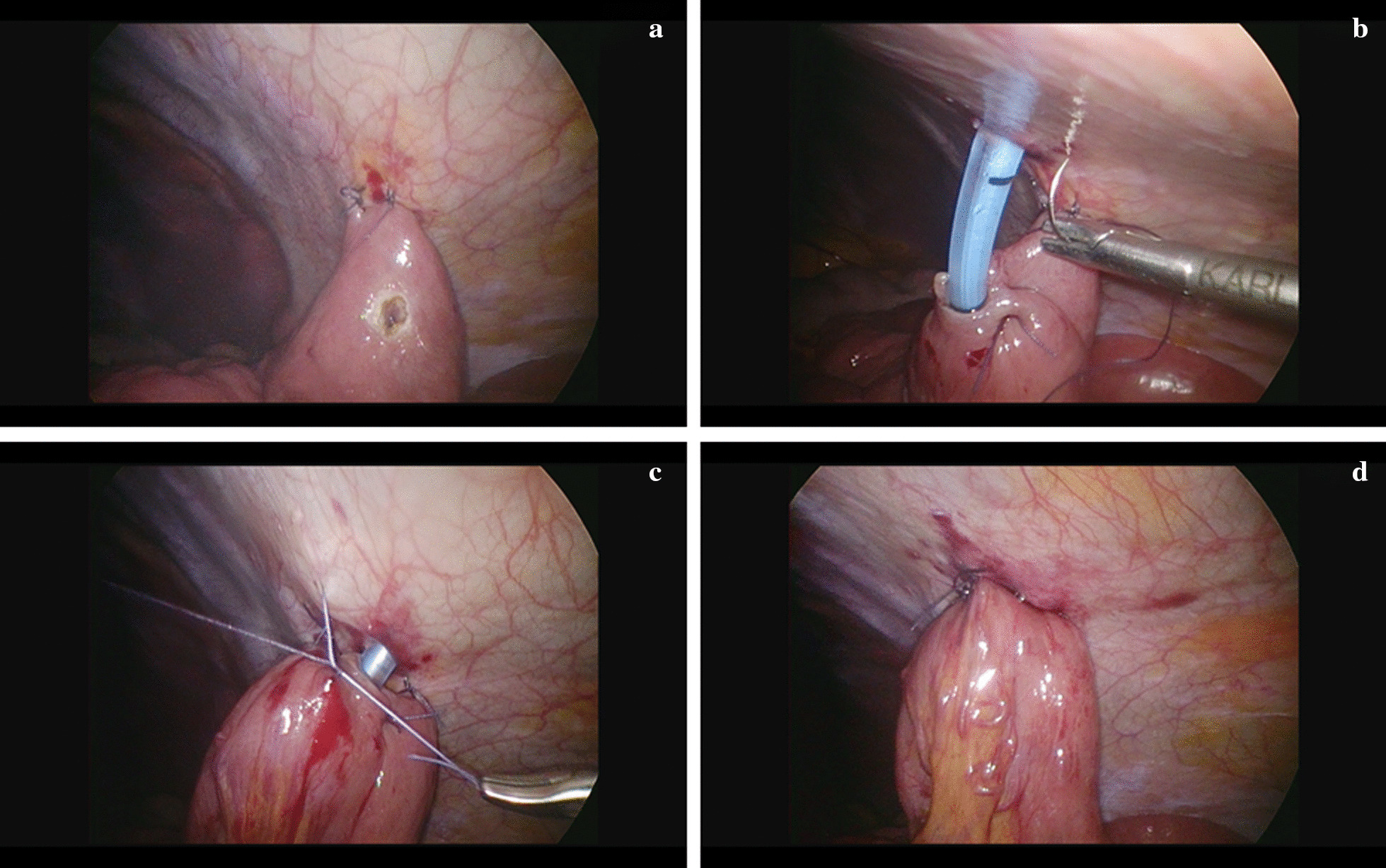
Stepwise procedures of laparoscopic feeding jejunostomy. **a** Two fixation stiches were made between the peritoneal surface of the cutaneous exit and the proximal jejunum, which was usually about 15 to 20 cm distal to the Treitz ligament. A small enterotomy was made by monopolar electrocautery at an appropriate location just opposite to the cutaneous exit. **b** A skin incision was made at the predetermined cutaneous exit and one 14Fr feeding tube was inserted through this incision into the distal jejunum. The tube was advanced to a designated length and one purse-string sero-muscular suture around the tube was performed. **c** Several more interrupted peritonization sutures were carried out to approximate the jejunum to the peritoneal side of the anterior abdominal wall. **d** The feeding tube was finally tested for patency and leakage after these procedures

For LJ, the patients were placed in the supine position. Most procedures were performed by two surgeons, with the index operating surgeon and camera man standing on the right side of the patient (Fig. 1a). The video laparoscope was introduced via a 5 mm vertical incision at about 3 cm below the umbilicus. Pneumoperitoneum was kept at 8 to 10 mmHg. Another 5 mm working port was created below the umbilical level at around left mid-clavicular line. The third 5 mm working port was created slightly above the umbilical level at right paramedian area (Fig. 1b). The peritoneal cavity was inspected carefully to confirm the patency of bowel loops and stages of the disease. Bowel graspers were introduced to lift the transverse colon upward and identify the Treitz ligament. The cutaneous exit of jejunostomy tube over the left abdominal wall was determined first. It should be located at about the midpoint of left costal ridge and umbilical line, with around an 8 to 10 cm distance to the two working ports. Two fixation stiches were then made between the peritoneal surface of this exit and the proximal jejunum, which was usually about 15 to 20 cm distal to the Treitz ligament (Fig. 2a). We preferred absorbable braided sutures such as 3–0 Vicryl (Ethicon Inc.) for purely laparoscopic jejunostomy. A bite over the peritoneal surface would be made first, followed by the bite over the sero-muscular layer of the selected jejunum. While securing the surgical tie, a manual pressure from the outside of the abdominal wall would be applied over the exit area to reduce the distance between the anterior abdominal wall and the jejunum. After securing these two stiches, the proximal jejunum should be anchored to the anterior abdominal wall. A small enterotomy was then made by monopolar electrocautery at an appropriate location just opposite to the cutaneous exit (Fig. 2a). A skin incision was made at this cutaneous exit and one 14Fr feeding tube was inserted through this incision into the distal jejunum. The tube was advanced to a designated length and one purse-string sero-muscular suture around the tube was performed (Fig. 2b). Several more interrupted peritonization sutures were then carried out to approximate the jejunum to the peritoneal side of the anterior abdominal wall (Fig. 2c). Similar manual pressure would be applied at each surgical tie. The feeding tube was finally tested for patency and leakage after these procedures (Fig. 2d). No drainage tubes were routinely placed.

For OJ, a vertical midline incision was made at upper abdomen at appropriate levels. The wound was deepened and the peritoneum was entered. The proximal jejunum was identified and a small enterotomy was made at about 15 to 20 cm distal to the Treitz ligament. One to two non-absorbable purse string sutures were placed around the enterotomy. A skin incision was then made at the predetermined cutaneous exit site, and one 14Fr feeding tube was inserted through this incision into the enterotomy. The tube was advanced to a designated length, and the jejunal loop around the tube was approximated and fixed to the peritoneal side of the cutaneous exit by multiple interrupted peritonization sutures made between the peritoneum and jejunum. The feeding tube was tested for patency and leakage after these procedures. No drainage tubes were routinely placed.

### Definition

Operation duration was defined as the time interval elapsed from anesthesia induction to extubation. The numeric rating scale (NRS) was adopted in our hospital to assess the degree of postoperative pain [20–24]. Late postoperative complications defined complications occurring 1 month after surgery and comprised catheter kinking requiring reintubation, poor wound healing/infection requiring wound debridement/repair or antibiotics treatment, catheter dislodgement requiring fluoroscopic intervention or reoperation, and ileus requiring prolonged fasting, parenteral nutrition, or operation. Cases with surgical mortality, defined as death within one month of surgery, were excluded from the current analyses.

### Statistics

The statistical analysis was performed with IBM SPSS Statistics 21 (IBM Corporation, Software Group, Somers, NY, USA). Fisher’s exact test or Pearson’s χ^2^ test was used to analyze categorical data. Student’s t test was used to analyze continuous variables. Statistical significance was defined as *P* values < 0.05 in two-sided tests.

## Results

There were 29 patients in the LJ group and 94 patients in the OJ group. The procedures were well tolerated in both groups. Table 1 summarized the patient demographics. As shown in the table, there were no significant differences in terms of patient gender, history of previous abdominal operations, preoperative albumin, preoperative hemoglobin level, body mass index (BMI), and performance status (ECOG) between the two groups (all *P* > 0.05). On the other hand, patients in the LJ group were significantly younger than patients in the OJ group (mean age 54.3 ± 9.5 vs. 61.6 ± 12.3, *P* = 0.004). As for the indications for surgery, malignancy remains the most common indication for jejunostomy, with more than 80% of patients in both groups undergoing operation due to underlying malignancy. Among malignancies, esophageal cancer was the most common type, followed by head/neck cancer and gastric cancer. In the meanwhile, it is noteworthy that 18.1% of OJ group had non-cancer related indications, compared to only 3.4% in the LJ group (*P* = 0.070).

**Table 1:** Patient demographics

N = 123	Laparoscopic	Open	*P* value
Patient number	29	94	
Gender (n (%))			0.731
Male	27 (93.1)	83 (88.3)	
Female	2 (6.9)	11 (11.7)	
Age^c^	54.3 ± 9.5	61.6 ± 12.3	0.004
Indications of jejunostomy			0.070
Cancer-related (n (%))	28 (96.6)	77 (81.9)	
Head/neck	7	25	
Esophageal	21	40	
Gastric	0	9	
Others	0	3	
Non cancer-related (n (%))	1(3.4%)	17(18.1%)	
Caustic injury	0	8	
Recurrent aspiration	1	7	
Others	0	2	
Previous abdominal operations (n (%))	1 (3.4%)	12 (12.8%)	0.297
Pre-OP albumin (mg/dL)^c^	3.41 ± 0.6	3.35 ± 0.7	0.694
Pre-OP hemoglobin (mg/dL)^c^	11.5 ± 2.1	11.3 ± 2.4	0.756
BMI^a^ (mg/dL)			
< 18.5 (underweight) (n (%))	10 (34.5)	31 (33.0)	1.000
18.5–25 (n (%))	19 (65.5)	63 (67.0)	
BMI (Mean ± SD)	19.9 ± 2.4	19.9 ± 3.7	0.969
ECOG^b^			0.449
0–2 (n (%))	28 (96.6)	85 (90.4)	
3–4 (n (%))	1 (3.4)	9 (9.6)	

^a^Body mass index

^b^Eastern cooperative oncology group

^c^Expressed as mean ± standard deviation

As for surgical variables, the LJ group had a significantly longer operation time than the OJ group (159 ± 57.2 mins vs. 128 ± 34.6 mins, *P* = 0.001). Only one patient underwent conventional open jejunostomy under local anesthesia. Forty-two patients (45.2%) in the OJ group underwent concomitant procedures, in contrast to only 5 patients (17.2%) in the LJ group (*P* = 0.008). No serious intraoperative complications were recorded in both groups (Table 2).

**Table 2:** Surgical variables of patients receiving feeding jejunostomy

N = 123	Laparoscopic (29)	Open (94)	*P* value
Operation duration (mins, mean ± SD)	159 ± 57.2	128 ± 34.6	0.001
Estimate blood loss, mL	5 ± 0	6.3 ± 4.1	0.081
Range	Minimal-5	1–20	
Anesthesia method	All GA^a^	1 LA^b^	
Catheter size	14 Fr	14 Fr	
Concomitant procedures (%)	5 (17.2%)	42 (45.2%)	0.008
Port-A	4	20	
Tracheostomy	1	5	
Enterolysis	0	13	
Others^c^	0	8	
More than 1 concomitant procedure	0	4	

^a^General anesthesia

^b^Local anesthesia

^c^Includes tumor excision/biopsy, umbilical/ventral hernia repair, etc.

Table 3 summarized the postoperative outcome following feeding jejunostomy. The pain scale on postoperative day 1 (POD1) was significantly lower in the LJ group than in the OJ group (*P* = 0.002). The implementation of early enteral feeding, i.e. feeding within 48 h of surgery, was comparable between the two groups (*P* = 0.216). The mean feeding time was around 2 days postoperatively in both groups (*P* = 0.525). The occurrence of late postoperative complications was minimal in both groups (*P* = 1.000). As for the application of chemotherapy, there was no significant difference in terms of date of chemotherapy initiation between the two groups. Both groups had started their chemotherapy at about 20 days after surgery (*P* = 0.950).

**Table 3:** Post-operative outcome of patients receiving feeding jejunostomy

N = 123	Laparoscopic (29)	Open (94)	*P* value
Immediate post-OP outcome			
POD1^a^ pain scale^bc^	2.03 ± 0.9	2.79 ± 1.2	0.002
Enteral feeding			0.216
≦ 48 h	25 (86.2)	70 (74.5)	
> 48 h	4 (13.8)	24 (25.5)	
Exact feeding time (day)	2.0 ± 0.5	2.27 ± 1.93	0.525
Late post-OP complication (n (%))	0 (0)	3 (3.2)	1.000
Catheter kinking	0	2	
Poor wound healing/infection	0	1	
Catheter dislodgement	0	0	
Ileus	0	0	
Post-OP chemotherapy (yes, n (%))	18 (62.1)	43 (45.7)	
Initiation date (POD)^c^	20.2 ± 15.1	19.9 ± 16.4	0.950
Initiation date (Range)	1–64	7–90	

^a^Postoperative day 1

^b^Assessed by numeric rating scale (NRS)

^c^Mean ± standard deviation

The mean duration of enteral feeding was 8.70 ± 2.00 months and 10.36 ± 1.69 months in the LJ and OJ groups, respectively (*P* = 0.610). More than 90% of the jejunostomies (93.1% in the LJ group and 92.6% in the OJ group, *P* = 1.000) were still functional after periods of feeding. Among these patients with functional jejunostomies, continued enteral feeding via jejunostomy was necessary in some patients due to persistent or progressive underlying disease while others may have their stomies closed after curative treatment was completed.

## Discussion

Laparoscopic surgery has been proven to be an effective surgical approach in many abdominal diseases, including acute cholecystitis, colon cancer, and gastroesophageal reflux disease [25–27]. With improvements in surgical techniques and laparoscopic instruments, laparoscopic surgery has also shown promising results in major abdominal operations in recent decades [28]. Laparoscopic feeding jejunostomy, however, did not attract much attention from surgeons worldwide. It may be due to the fact that feeding jejunostomy by nature is not a complex or important procedure. Surgeons would not spend their time investigating the benefits of an insignificant operation. We, in contrast, hold the opposite opinion. Since enteral feeding is superior to parenteral nutrition, feeding jejunostomy should be indicated for patients intolerant to per oral feeding. Given inherent merits of smaller incisions, better cosmesis, less postoperative pain, and earlier recovery, laparoscopic feeding jejunostomy should be the preferable approach and thus cannot be overlooked! In the current study, we described our method of purely laparoscopic feeding jejunostomy and compared the result with that of conventional open counterpart. Our study is thus one of the first in the English literature to imply the advantages of laparoscopic feeding jejunostomy over the open ones. Since all of the LJ in the present study were conducted by one single surgeon, we also believe the results should be more homogeneous and reliable.

Our purely laparoscopic feeding jejunostomy possesses plenty of advantages. First, we demonstrated that the LJ group did have significantly less pain than the OJ group, which although was not a surprising finding but rarely described [29, 30]. Secondly, the postoperative recovery, in terms of enteral feeding, was not compromised by the laparoscopic approach. More than 85% of patients in the LJ group started enteral feeding within 48 h of surgery, which was similar to previous studies [30]. The initiation of chemotherapy in cancer patients was also comparable between LJ and OJ. Our findings indicated that the laparoscopic procedure can not only reduce the postoperative pain but also maintain the intrinsic effectiveness of conventional open approach.

Next, in the current laparoscopic procedure, we used 3-0 Vicryl, instead of V-loc barbed suture, for the intracorporeal sutures. Since it has been described that the V-loc sutures may cause small bowel obstruction or ileus, our choice of 3-0 Vicryl may thus reduce the risk [31–33]. One may argue that it would be very challenging to secure the stich over the peritoneal side of the anterior abdominal wall without the aids of fancy suturing instrument such as V-loc barbed suture [30]. We believe this obstacle can be overcome by our technique of external manual pressure and pneumoperitoneum of 8 to 10 mmHg. Furthermore, we employed three 5 mm trocar ports, instead of 10–18 mm ports, in the current procedure [10]. It would definitely reduce the scar and postoperative pain. Last but the least, through these repetitive intracorporeal sutures and ties, younger surgeons can excel their laparoscopic suturing techniques before advancing to more complicated intra-abdominal operations.

Since its first introduction by Gauderer et al., percutaneous endoscopic gastrostomy (PEG) has become one of the most popular routes of nutritional support worldwide [34, 35]. Due to lower cost, less need for general anesthesia and less invasive nature, PEG has been considered as a better choice for enteral feeding over the surgical methods. Nevertheless, complications, which range from minor ones such as wound infection, gastric outlet obstruction, and peritonitis to major complications including bleeding, bowel perforation, and necrotizing fasciitis, do occur after PEG [34]. Laparoscopic jejunostomy, in contrast, was found to have few complications based on the current study. Additionally, PEG is inappropriate in patients with gastric or duodenal malignancies. Moreover, PEG requires specialized endoscopist, radiologists, custom-made feeding tubes, and fixations devices to securely complete the procedure. The replacement of PEG tubes, unlike the LJ tubes, also requires special equipment. Last but not the least, LJ provides opportunity to perform thorough abdominal exploration in the first place, which can assess the extent of disease prior to the initiation of chemotherapy. The inherit merit of laparoscopic surgery to reduce post-operative adhesions could also facilitate the following curative surgery. Given these potential benefit over PEG, we believe laparoscopic jejunostomy should become a fair alternative to PEG in hospitals where PEG techniques or facilities are not available.

Despite our remarkable findings, the current study still had several drawbacks. First, the two groups were not homogeneously distributed. There seemed to be selection bias when performing the procedure. The LJ group was much younger and received less prior abdominal operations. There was also less caustic injury in the LJ group, and more patients in the OJ group underwent concomitant procedures. We believe there are some explanations: Since the index surgeon is a dedicated surgical oncologist, while the surgeons who perform the OJ also include general surgeons, the distribution of patient population may thus be heterogeneous. Next, younger patients were expected to have less comorbidities and better cardiopulmonary reserve, who may thus sustain better under pneumoperitoneum. The history of prior abdominal operations and the necessity of thorough gastrointestinal tract evaluation in cases with caustic injury may render surgeons less willing to perform the operation under laparoscopy. As for concomitant procedures, the frequency of concomitant extra-abdominal procedures was not that diverse between the two groups (17.2% and 26.6% in the LJ and OJ groups, respectively). Nevertheless, we still believe a well-designed randomized study with matched variables is warranted to investigate the actual effect of laparoscopic feeding jejunostomy.

Secondly, the operation duration was about 30 min longer in the LJ group in the current study. This prolonged operation time was due to our initial inexperienced technique. After excluding the first two cases, the mean operation duration would reduce to 146.8 min, 158 min faster than the initial experience. We believe the operation duration would be even shorter when the learning curve is overcome. Next, there is still a discrepancy between our operation duration and those of previous research [4, 10, 13–15, 17–19, 30, 36–39]. We believe this is due to different definitions regarding operation time. Finally, the sample size is too small; future larger scale studies with matched variables are thus warranted to validate our findings.

## Conclusion

In conclusion, the current study demonstrated that our purely laparoscopic feeding jejunostomy is a safe and feasible procedure with less postoperative pain and excellent postoperative outcome. In addition, it provides surgeons opportunities to enhance intracorporeal suture techniques. Therefore, we believe, in selected patients such as those of younger age, with better performance status, or without prior abdominal operations, laparoscopic feeding jejunostomy should become a standard surgical procedure and should not be neglected. Further large scale prospective studies are warranted to validate our findings.

## Data Availability

All data generated or analyzed during the study are included in this published article. Raw data may be requested from the authors with the permission of the institution.

## Abbreviations

BMI: Body mass index
CGMH: Chang Gung Memorial Hospital
ECOG: Eastern Cooperative Oncology Group
Hr: Hour
IRB: Institutional review boards
LA: Laparoscopic appendectomy
LC: Laparoscopic cholecystectomy
LG: Laparoscopic gastrectomy
LH: Laparoscopic hepatectomy
LJ: Laparoscopic jejunostomy
LP: Laparoscopic pancreatectomy
IRB: Institutional review boards
NRS: Numerical rating scales
OJ: Open jejunostomy
POD: Post-operative day
PEG: Percutaneous endoscopic gastrostomy
